# Effect of Natural *Commiphora myrrha* Extract against Hepatotoxicity Induced by Alcohol Intake in Rat Model

**DOI:** 10.3390/toxics10120729

**Published:** 2022-11-26

**Authors:** Abeer S. Alahmari, Haitham I. El-Mekkawy, Amin A. Al-Doaiss, Manal A. Alduwish

**Affiliations:** 1 Biology Department, Faculty of Science, King Khalid University, Abha 61413, Saudi Arabia; 2 Research Center for Advanced Materials Science (RCAMS), King Khalid University, Abha 61413, Saudi Arabia; 3 Anatomy and Histology Department, Faculty of Medicine, Sana’a University, Sana’a 1247, Yemen; 4 Department of Biology, Faculty of Science and Humanities, Prince Sattam Bin Abdulaziz University, Alkarj 11942, Saudi Arabia

**Keywords:** *Commiphora myrrha*, ethanol, hepatic injury, liver function, oxidative stress

## Abstract

The oral intake of alcohol has become a widespread concern due to its high risk to body health. Therefore, our purpose in this study was to reveal the antioxidant efficacies of natural *Commiphora myrrha* on hepatotoxicity and oxidative stress induced by ethanol in adult male rats, especially because these were not adequately revealed by previous studies. We examined the impacts of *C. myrrha* in male Sprague Dawley rats orally treated with *C. myrrha* (500 mg/kg) alone or in combination with 40% ethanol (3 g/kg), daily for 30 days. The results showed that treatment with *C. myrrha* after the oral consumption of ethanol caused a reduction in serum liver function parameters (alanine transferases, aspartate transaminase, and total bilirubin), hepatic tumor markers (α-L-flucosidase and arginase), and hepatic lipid peroxidation indicator (thiobarbituric acid reactive substances), as well as a slight restoration (not significant) in the levels of superoxide dismutase, catalase, reduced glutathione; and total antioxidant capacity. In addition, it alleviated histopathological changes in the liver, as revealed by decreased areas of inflammatory infiltrate, milder necrosis, and noticeably reduced periportal fibrosis and hemorrhage. The therapeutic efficiency of *C. myrrha* could be due to its rich sesquiterpenoids content which possesses anti-inflammatory properties and ROS-scavenging activities. Our findings provide evidence that the attenuation of oxidative stress by *C. myrrha* enables hepatic tissue to suppress inflammatory and oxidative mechanisms, resulting in enhanced liver structure and function. Therefore, *C. myrrha* extract shows promise as a protective and therapeutic supplement against toxic agents.

## 1. Introduction

The liver is the primary target organ exposed to many foreign substances before dilution in the systemic circulation because it is an entry portal to the circulatory system and the other tissues of the body. It plays a critical role in determining the toxicity of various harmful agents due to its major role in the metabolism, transmission, and elimination of toxic substances. Therefore, it is likely to be affected by various toxic agents known to cause damage to liver tissues [1]. For this reason, hepatocyte functions are usually evaluated to examine toxicities [2].

According to a World Health Organization (WHO) report in 2018, about 3.3 million annual deaths worldwide can be regarded as being caused by alcohol intake [3]. Subsequently, on 24–29 January 2022, the WHO submitted a draft plan of action (2022–2030) to implement international strategy to decrease the harmful use of alcohol as a public health matter [4]. The main kind of alcohol that is consumed is ethanol. Within the gastrointestinal tract, the majority of ethanol (~70%) is absorbed by the small intestine via diffusion and then passes into the liver through the portal vein. A small amount of ingested ethanol is absorbed by the stomach (~20%) [5,6]. In the hepatocytes, ethanol is metabolized through the alcohol dehydrogenase (ADH) system via nicotinamide adenine dinucleotide (NAD+) as a coagent [7] in three main stages: first, oxidation of ethanol to acetaldehyde; second, metabolism of acetaldehyde to acetate; finally, catabolism of acetate to CO_2_ and H_2_O [8].

As a result of ethanol metabolism, ethanol and its metabolites induce direct hepatotoxicity by activation of innate immune functions and secretion of proinflammatory cytokines and chemokines [9]. These mechanisms lead to the destruction of the mitochondria and the microtubule structure of hepatic cells [7]. Ethanol also plays a role in the stimulation of Kupffer cells, which are involved in the production of several soluble factors such as reactive oxygen species (ROS) and factor nuclear kappa B (NF-κB) [10]. These agents act to stimulate hepatic stellate cells (HSCs), a major step in liver injury and fibrosis, by the promotion of collagen synthesis and accumulation of extracellular matrix proteins [11]. Consequently, liver destruction occurs with severe histopathological changes, such as cell damage, fatty infiltration, and fibrosis [12].

In addition, ROS are linked to the enhancement of oxidative stress and inflammation in the hepatic tissues, as well as triggering the release of liver enzymes, including aspartate transaminase (AST), alanine transaminase (ALT), and alkaline phosphatase (ALP), into the blood [13]. On the other hand, ethanol stimulates the expression of cytochrome P450 2E1 (CYP2E1) in the hepatocytes, which in turn induces dysregulations in lipid peroxidation by ROS free radicals. Therefore, it damages antioxidant genes and causes a dynamic imbalance of the antioxidant systems, such as catalase (CAT), superoxide dismutase (SOD), glutathione (GSH), and glutathione peroxidase (GPx), which have a crucial impact in ROS scavenging [14]. Therefore, a functional diet that has antioxidant effects could be a curative agent for attenuating alcohol toxicity.

Several experimental studies on animal models have demonstrated that various medicinal plants exhibit protective activities and can prevent various diseases through their ability to scavenge free radicals and increase the expression of antioxidants [15]. *Commiphora myrrha* is one of the most famous medicinal plants and one of the most highly valued natural products in Arabia, India, and certain regions in Africa [16]. It has been used throughout history to make incense and as a perfume. It also has been tightly linked to female health and purifying rites in Bible times [17]. The ancient Egyptians used *C. myrrha* to embalm mummies [18]. It contains several inorganic components including chlorine, magnesium, chromium, calcium, aluminum, and phosphorus, along with the presence of a significant number of organic constituents such as limonene, beta selinene, curzerene, and beta-element [19]. Its gum resin has several therapeutic applications in the management of wound healing, swellings, and inflammation [20]. Pharmacologic studies have demonstrated that *C. myrrha* has various medicinal benefits due to its contents that act as antitumoral [21], antiparasitic [16], anti-inflammatory, analgesic [20], antioxidant, and antinociceptive [22] agents. According to previous studies, myrrh has the ability to lower the synthesis of proinflammatory mediators as well as promote internal antioxidant capacity, which leads to an improvement in the symptoms of inflammation in rats [23]. Additionally, myrrh is a cytotoxic agent with anti-tumor activity against several types of cancers [21].

Because the antioxidant potential of *C. myrrha* with regard to complications of ethanol intake, such as liver dysfunction, has not been widely studied, we conducted this study to evaluate the probable effects of natural *C. myrrha* extract on hepatic injury induced by ethanol in adult male rats.

## 2. Materials and Methods

### 2.1. Ethics Statement

The current experimental protocol was approved by the Research Ethical Committee of King Khalid University, Saudi Arabia (approval No. 2022–2125), and carried out in accordance with the associated guidelines.

### 2.2. Drug and Chemicals

We purchased oleo-gum of *C. myrrha* from a commercial market in Khamis Mushait, Saudi Arabia, in the form of brownish masses. Its original source was the western area of Saudi Arabia. All chemicals and reagents (Saudi Chemical Co, Abha, Saudi Arabia) were of the highest analytical grade, available in the laboratories of the biology department at King Khalid University.

### 2.3. C. myrrha Extract Preparation

We washed fresh pieces of *C. myrrha* with distilled water to eliminate adhering dust, which we then crushed into small parts. We mixed approximately 10 g of these parts with 200 mL of deionized water in a flask, which we then placed in a water bath at 80 °C for 7 h. We centrifuged the resulting solution at 4000× *g* for 15 min, then filtered it through filter paper and kept it at 4 °C until use [24].

### 2.4. Phytochemical Analysis of C. myrrha Extract Using Gas Chromatography-Mass Spectrometry (GC–MS)

Phytochemical analysis of *C. myrrha* extract was conducted using GC/MS to determine the nature of the extract components which might contribute to the protective impacts against ethanol toxicity according to the method described by Alqahtani and others [25]. 

### 2.5. Animals and Work Design

We obtained 28 Sprague Dawley rats (adult males, weighing 200 ± 50 g) from the animal house of the Science College at King Khalid University (Abha, Saudi Arabia). We acclimatized the animals for two weeks in propylene cages with sawdust bedding in an animal house with a light/dark cycle at normal temperature (22 ± 2 °C). They received a balanced ad libitum diet and tap water. We considered and avoided all animal stress factors to the greatest extent possible.

After acclimatization, we randomly divided the rats into four groups (*n* = 7 per group). All rats were orally treated by gastric gavage daily for 30 days as follows:

- Group 1 (control): rats were not given any drugs throughout the experiment, as a control group.
- Group 2 (*C. myrrha* extract): rats received 500 mg/kg of *C. myrrha* extract [25].
- Group 3 (ethanol): rats were treated with 40% ethanol (3 g/kg) [26].
- Group 4 (ethanol + *C. myrrha* extract): rats were administered 40% ethanol (3 g/kg), followed by *C. myrrha* extract (500 mg/kg) after two hours.

The dose of *C. Myrrha* extract was chosen according to the preliminary experiments in order to verify the safety of this treatment on liver tissue without side effects. Additionally, the dose of ethanol was determined based on the preliminary experiments to ensure its ability to cause hepatotoxicity without causing death in rats. Detailed preliminary experiments are provided in Supplementary Materials.

### 2.6. Sampling

Twenty-four hours after the final treatments, we sacrificed all animals under light ether anesthesia. We obtained blood samples from a heart puncture to prepare sera, which we then stored at −80 °C until use. We excised liver samples, which we then washed with cold normal saline 0.9%. We then divided each liver into two parts for biochemical and histopathological examination. For biochemical analysis, we homogenized the liver in 100 mM phosphate-buffered saline (PBS) with 1 mM EDTA, pH 7.4, which then centrifuged at 12,000× *g* for 25 min at 4 °C. Prior to use, we kept the supernatant at −80 °C.

### 2.7. Biochemical Analysis

#### 2.7.1. Evaluation of Liver Function

We measured the activities of AST and ALT and the concentration of total bilirubin in serum using diagnostic kits (Biodiagnostic, Giza, Egypt) following the manufacturer’s instructions. AST and ALT were measured, as described by Reitman and Frankel [27], while total bilirubin concentration was measured, as described by Westwood [28].

#### 2.7.2. Estimation of Liver Tumor Markers

For diagnosis of the early onset of hepatocellular carcinoma and cellular injury, we determined serum α-L-fucosidase and arginase parameters using commercial colorimetric assay kits (Biodiagnostic, Giza, Egypt) in accordance with the manufacturer’s protocol. α-L-fucosidase assay is based on the splitting of 4-nitrophenol from the synthetic substrate. After adding the stop solution, the color of nitrophenol becomes intense. The absorbance of the resultant intense nitrophenol was measured at 404 nm. There is a direct proportion between the elevation in absorbance at 404 nm and the enzyme activity [29]. While arginase assay is based on the hydrolysis of arginine to ornithine and urea. Urea is colorimetric determined in an acid medium at 525 nm [30].

#### 2.7.3. Determination of Lipid Peroxidation

As a marker of lipid peroxidation, we measured the level of hepatic thiobarbituric acid reactive substances (TBARS) according to the method of Rael [31] using TBARS Assay Kit (Biodiagnostic, Giza, Egypt). This assay is based on the reaction of thiobarbituric acid with TBARS to produce a pink-colored substance. At 535 nm, the color intensity is directly proportional to TBARS concentration in the hepatic samples.

#### 2.7.4. Assay of Antioxidant Activity

To evaluate the response to cellular stress, we measured the activities of SOD, CAT, GPx, GSH, and total antioxidant capacity (TAC) in the liver by colorimetric assays using commercially available kits (Biodiagnostic, Giza, Egypt) according to Vinatier et al. [32].

### 2.8. Liver Histopathological Examination

We immediately fixed the second part of the fresh liver of all animals in 10% formalin after collection. We processed samples, which we then dehydrated in graded series of alcohol, cleared in two changes of xylene, infiltrated in three changes of melted paraffin wax, embedded in paraffin, and cut into sections 4–5 μm thick using a rotary microtome. We then mounted sections on slides, which we then stained with hematoxylin and eosin (H & E) and Masson trichrome for microscopic investigation. We captured digital images using an Olympus microscope. Histopathological alterations including lymphocyte infiltration, hemorrhage, necrosis, and fibrosis were scored by estimating 15 microscopic fields of liver sections using a scoring checklist: (0 = none, 1 = mild, 2 = moderate, 3 = severe) as described by Damiano et al. [33]. 

### 2.9. Statistical Analysis

The numerical data are expressed as mean ± standard error (SE). We assessed statistical differences between experimental groups using one-way ANOVA by SPSS 16 statistical software (Chicago, IL, USA). By using Tukey’s multiple range test procedure, we set *p* < 0.05 as indicating a statistically significant difference. The distribution differences of the histopathological semiquantitative alterations among groups were evaluated using the Kruskal–Wallis H test, followed by Dunn’s post hoc tests at *p* < 0.05.

## 3. Results

### 3.1. Chromatographic Analysis (GC–MS) of C. myrrha Extract

The phytochemical contents along with the respective percentage in the myrrh extract are illustrated in Table 1. The GC-MS analysis revealed the presence of 25 components representing 95.96% of the extract. The *C. myrrha* extract was rich in sesquiterpenes, and the most predominant component was curzerene which represented 31.07% of *C. myrrha* extract. Other sesquiterpenes include aromadendrene 2 (2.07%), germacrene b (1.96%), gamma-elemene (0.84%), germacrene-D (0.56%), β-Elemene (0.12%), etc. We also detected other major content including hepta-2,6-dienoic acid (18.97%), 3A,4,4A,7,7A,8,9,9A-octahydro-1,4,8-metheno-1H-cyclopent[f]azulene (13.20%), and alpha thejenal (12.13%).

### 3.2. Effects on Liver Function Parameters

**Figure 1:**
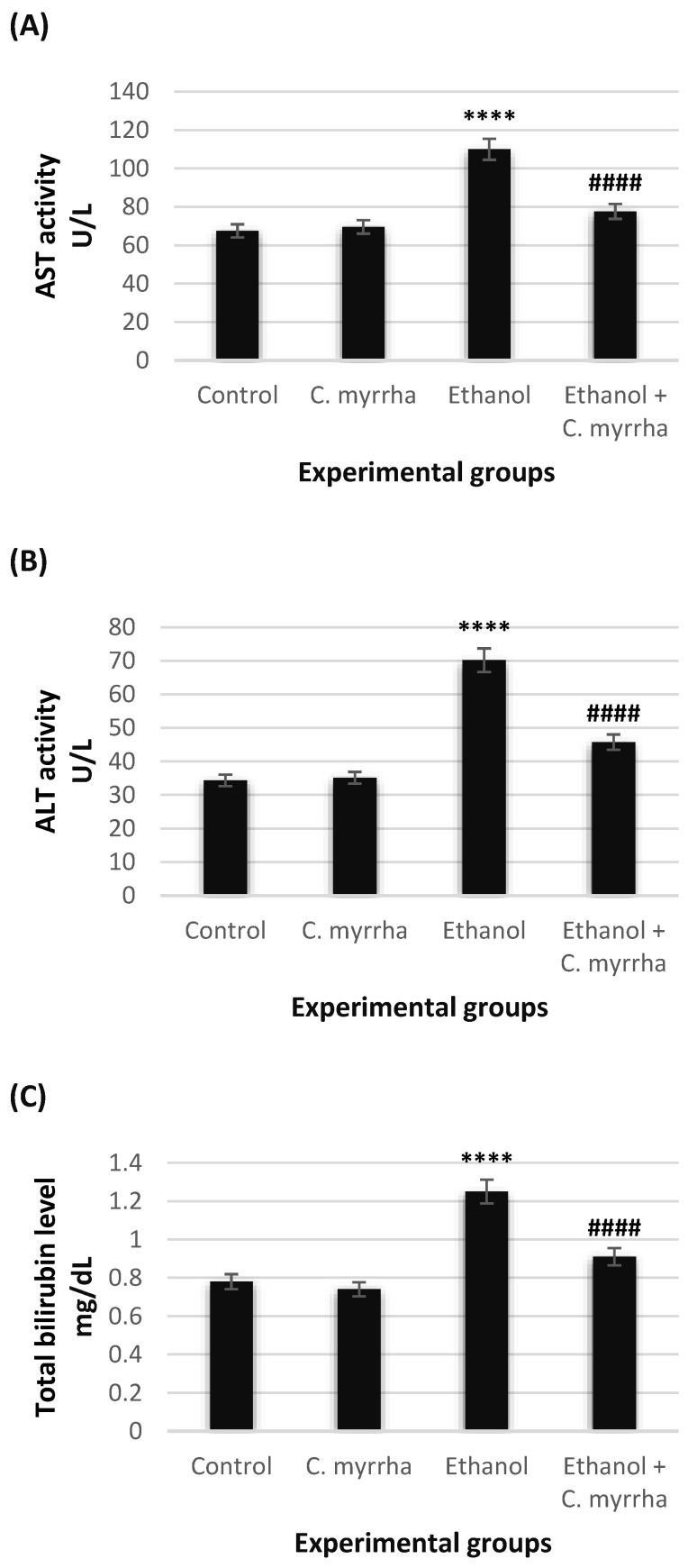
Effects of *C. myrrha* extract (500 mg/kg) and/or ethanol (40%, 3 g/kg) intake on the liver function parameters in serum after 30 days of treatment. (**A**) Aspartate transaminase (AST) activity expressed as units per liter (U/L). (**B**) Alanine transaminase (ALT) activity expressed as units per liter (U/L). (**C**) Total bilirubin level is expressed as milligrams per deciliter (mg/dL). All values are expressed as mean ± standard error (SE) of *n* = 7. **** *p* < 0.0001 vs. control; ^####^ *p* < 0.0001 vs. ethanol-treated group.

The hepatic function parameters we tested in this experiment were AST, ALT, and total bilirubin (Figure 1). In group 2, which received *C. myrrha* extract only, we recorded no significant differences in the values of AST, ALT, or total bilirubin (*p* > 0.05) in comparison with group 1 (control). We found highly significant elevations in AST, ALT, and total bilirubin levels (109.96 U/L, 70.17 U/L and 1.25 mg/dL, respectively; *p* < 0.0001) in group 3 rats after they were treated with 40% ethanol only compared with the control group. However, cotreatment with *C. myrrha* extract significantly reduced the adverse effects of ethanol at the end of treatment, with significantly lower (*p* < 0.0001) AST, ALT, and total bilirubin values measured in the serum of 77.58 U/L, 45.73 U/L and 0.91 mg/dL, respectively.

### 3.3. The Hepatic Tumor Markers

**Figure 2:**
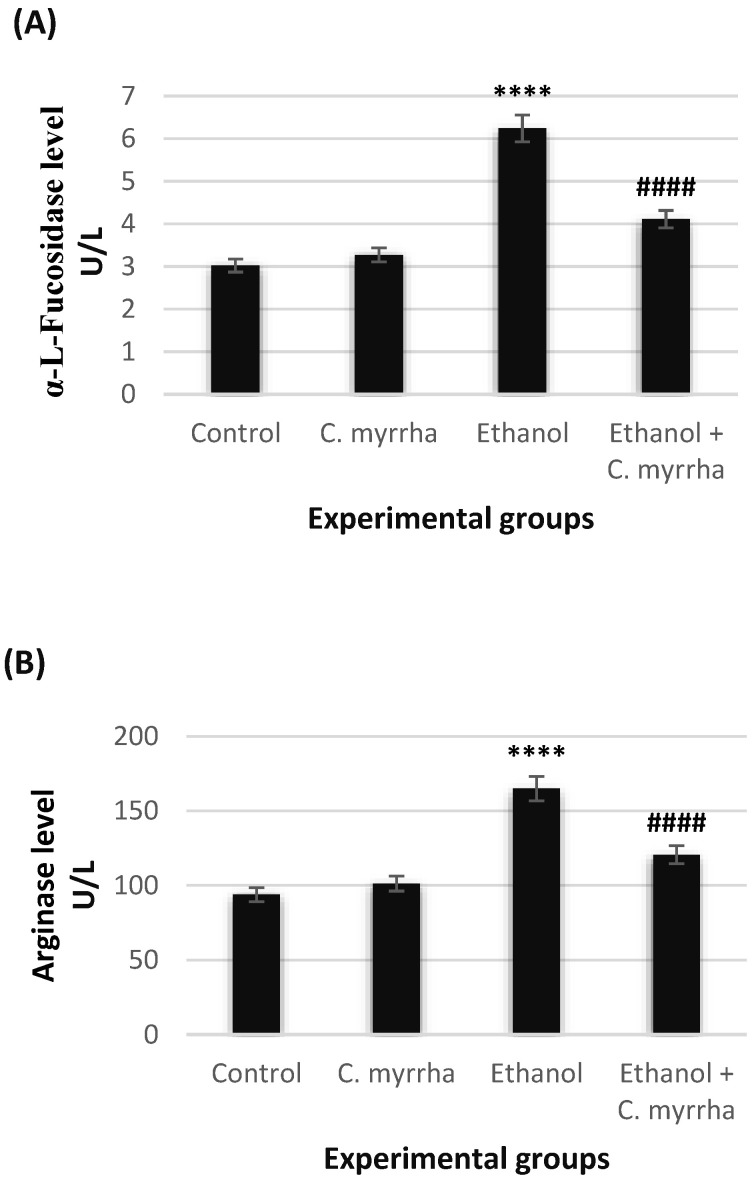
Effects of *C. myrrha* extract (500 mg/kg) and/or ethanol (40%, 3 g/kg) on hepatic tumor markers in serum after 30 days of treatment. (**A**) α-L fucosidase level expressed as units per liter (U/L). (**B**) Arginase level expressed as units per liter (U/L). All values are expressed as mean ± standard error (SE) of *n* = 7. **** *p* < 0.0001 vs. control; ^####^ *p* < 0.0001 vs. ethanol-treated group.

Serum α-L-flucosidase and arginase levels significantly increased (*p* < 0.0001) in group 3, which received 40% ethanol only (6.24 ± 0.39 and 164.95 ± 5.05 U/L, respectively) compared with group 1 (control) (3.02 ± 0.20 and 93.86 ± 4.17 U/L, respectively). However, coadministration with *C. myrrha* extract in group 4 significantly decreased (*p* < 0.0001) both marker levels in the serum to 4.11 ± 0.33 and 120.58 ± 4.21 U/L, respectively. When *C. myrrha* extract was used alone in group 2, we observed no significant changes in either α-L-flucosidase or arginase levels (3.27 ± 0.33 and 101.27 ± 4.07 U/L, respectively) compared with the control (Figure 2).

### 3.4. Effect on Lipid Peroxidation Biomarker

We estimated lipid peroxidation in the hepatic tissue by measuring the TBARS concentration. As shown in Table 2, we observed no significant (*p* > 0.05) change in TBARS value among the experimental rats in group 2 after treatment with *C. myrrha* extract only for 30 days compared with the control value in group 1. In contrast, the ethanol intake in group 3 resulted in remarkable increases in TBARS level (*p* < 0.0001). However, cotreatment with *C. myrrha* extract in group 4 led to a significant improvement in this biomarker (*p* < 0.0001) in comparison with group 3.

### 3.5. Activity of Antioxidant Parameters

The values of the antioxidant parameters (SOD, CAT, GSH, GPx, and TAC) in the hepatic tissues obtained from the rats in all experimental groups after 30 days of treatment are illustrated in Table 2. The values of SOD, CAT, GSH, GPx, and TAC after being treated with *C. myrrha* extract (group 2) were still close to those of the control group in group 1 (*p* > 0.05). However, the values of all these parameters were significantly lower in the livers of rats treated with 40% ethanol only (*p* < 0.0001) in comparison with the control. Cotreatment with *C. myrrha* extract in group 4 rats resulted in a slight restoration in SOD, CAT, GSH, GPx, and TAC values, but not significantly (*p* > 0.05), compared with those of group 3 rats, except in the case of GPx (*p* < 0.05).

### 3.6. Histopathological Evaluations

**Figure 4:**
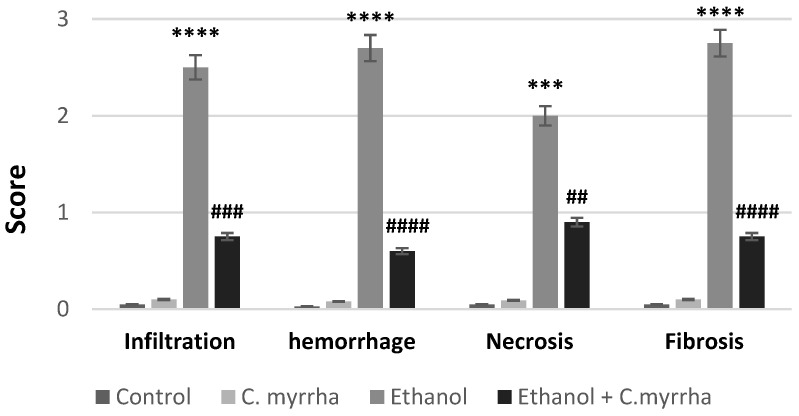
Semi-quantitative histopathological evaluation of the hepatic tissues from all experimental groups illustrating severity scores of lymphocyte infiltration, hemorrhage, necrosis, and fibrosis for experimental groups. *** *p* < 0.001; **** *p* < 0.0001 vs. control; ^##^ *p* < 0.01; ^###^ *p* < 0.001; ^####^ *p* < 0.0001 vs. ethanol-treated group.

The observations and the semi-quantitative histopathological evaluation of the hepatic tissues from all experimental groups are shown in Figure 3I Hematoxylin and eosin (H & E) stain, Figure 3II Masson trichrome stain, and Figure 4 Semi-quantitative histopathological evaluation. The liver of control group rats (group 1) shows a normal architecture of lobule structures with a normal distribution of collagen fibers around the central vein, in sinusoidal spaces, and between parallel cords of hepatocytes (Figure 3(A_I_) and Figure 3(A_II_)). Upon analysis, the hepatic tissue of *C. myrrha* extract-treated group rats (group 2) showed an apparently intact architecture similar to that of the control group with no significant histopathological changes (Figure 3(B_I_) and Figure 3(B_II_)). In contrast, ethanol-treated group rats (group 3) illustrated histopathological changes in the hepatic tissue, including dilation of congested central vein, lymphocyte infiltration (*p* < 0.0001), sinusoid hemorrhage (*p* < 0.0001), necrosis (*p* < 0.001), and edematous area around the blood vessels at the portal space (Figure 3(C_I_–E_I_)). Additionally, ethanol-treated group showed a high deposition of collagen fibers (blue) around the central vein, in addition to dilation and congestion of the portal vein with portal spaces expanded by abundant fibrous tissues as a marker for severe periportal fibrosis (*p* < 0.0001), and dilation of central veins associated with dilation of blood sinusoids (Figure 3(C_II_,D_II_)). Cotreatment with ethanol + *C. myrrha* extract (group 4) showed improvement in the histopathological alterations in hepatic tissues, as revealed by decreased areas of inflammatory infiltrate (*p* < 0.001), milder necrosis (*p* < 0.01), and noticeably reduced periportal fibrosis (*p* < 0.0001), congestion, and hemorrhage (*p* < 0.0001) compared to ethanol-treated group (Figure 3(F_I_) and Figure 3(E_II_)).

## 4. Discussion

Oral alcohol intake is a widespread health problem due to its high risk to body health; therefore, in the present study, we investigated the in vivo protective activity of *C. myrrha* extract as a natural product with respect to alcohol-induced hepatotoxicity in rats. We sought evidence of improved liver function and structure through an analysis of markers of hepatic function, oxidants, and antioxidants; as well as by means of histopathological examinations, as the role of this natural extract in relieving the deleterious effects of alcohol on the liver is not yet fully understood.

Our data illustrated that the administration of 40% ethanol noticeably increased the hepatic function parameters and hepatic tumor biomarkers. This hepatotoxic effect of ethanol was confirmed by the histopathological changes in the hepatic tissue. Our findings are compatible with the results of several studies on rat models [34]. According to previous research, ethanol-induced oxidative stress in the hepatocytes plays the main role in the development of hepatotoxicity and liver dysfunction [35]. In addition, the elevated levels of serum AST, ALT, and total bilirubin are signs of increased permeability, damage, and necrosis of hepatocytes [34]. On the other hand, several studies demonstrated that the high level of hepatic tumor markers may be an indicator for liver injury and reflect mechanisms of cellular injury [36]. So, we thought that measuring them at this time (the 30 days of the experiment) might give us early evidence of the onset of a tumor and cellular damage.

Our study has shown a fat accumulation in liver tissue in the histological sections obtained from rats treated with ethanol, with an elevation in TBARS level as a marker for lipid peroxidation at the same time. Evidence suggested that ethanol intake could raise the amount of TBARS in the liver tissues, enhance lipid peroxidation, and fail to block excessive free radical molecules, which would stimulate oxidative stress [37]. Our observations may be due to the formation of acetaldehyde as a metabolite of ethanol that induces the destruction of fatty acid β-oxidation in mitochondria to stimulate the synthesis of fatty acid, which causes an accumulation of fat in the hepatocytes as illustrated in previous research [38]. We also observed the appearance of severe periportal fibrosis in the liver tissue after 30 days of ethanol treatment. According to previous studies consistent with our results, this may be because drinking alcohol promoted the permeability of the intestine allowing lipopolysaccharide (LPS) to pass into the liver by the portal vein. The LPS is known to stimulate Kupffer cells to release cytokines, which in turn promote stellate cells to produce the extracellular matrix, mainly collagen, which results in liver fibrosis [39].

In addition, our results indicated lymphocyte infiltration after ethanol intake. This result was consistent with Elaby and Ali [40]. The negative effect of ethanol may be due to the fact that metabolites of ethanol expose hepatocytes to oxidative stress and apoptosis. Therefore, Kupffer cells release a variety of inflammatory agents, including cytokines, chemokines, and damage-associated molecular patterns (DAMPs), which in turn are involved in stimulating inflammation and immune responses, that enhance the activation and infiltration of innate immune cells [41]. Several scientific studies suggested that there is a correlation between hepatic inflammation and ROS production [42]. Inflammatory process and oxidative stress often alternately affect each other; ROS produced from injured cells stimulate inflammatory cells, and the stimulation of these inflammatory cells further promotes oxidative stress by releasing ROS and reactive nitrogen species (RNS) such as nitric oxide and peroxynitrite [43]. Moreover, ethanol and its metabolites result in impaired regulation of lipid peroxidation via ROS, which inhibits the genes expression of the antioxidants causing dysfunction and imbalance in antioxidant systems such as SOD, CAT, GPX, and GSH [44], as noted in our results after ethanol consumption.

Several phytonutrients are known as perfect antioxidants that have a preservative impact against the oxidative injury induced by toxic agents [45]. Among those nutrients is *C. myrrh,* which is known for its effectiveness in treating different diseases, especially in Arab societies [46]. In the current study, we dosed animals with *C. myrrha* extract to detect any side effects that may result from its consumption. Our results revealed that this extract is safe for the liver organ by measuring certain parameters such as liver function biomarkers, lipid peroxidation indicator, and antioxidants, and finding no significant differences compared with the control group. Seifried et al. reported that the consumption of natural antioxidant products in certain doses has more benefits with fewer adverse impacts [47].

To investigate its effectiveness against ethanol toxicity, we administered the animals with ethanol + 500 mg/kg of *C. myrrha* extract (group 4). Data in the present study showed that treatment with *C. myrrha* extract in combination with ethanol (group 4) lead to a decline in ALT and AST, total bilirubin, α-L-flucosidase, and arginase values, as well as improvement in the histopathological alteration in hepatic lobules. These results confirm that *C. myrrha* was able to inhibit hepatotoxic features induced by ethanol intake. Our results have been confirmed in numerous studies of the protective effect of *C. myrrha* extract against several toxic agents [23,48,49]. Alqahtani et al. demonstrated that myrrh extract can diminish disorders of liver function biomarkers in proportion to levels of concentration, due to the presence of natural bioactive components in the extract, which may be able to attenuate hepatic damage caused by free radicals [50]. In addition, Chen et al. reported that *C. myrrha* has the ability to arrest the proliferation of hepatocarcinogenesis in a dose-dependent manner [51]. The useful impacts of *C. myrrha* in our study may be related to its antioxidant activities, which provide a key contribution to the hepatoprotective process through the removal of free radicals and restoration of antioxidant defense activity inside the body, as illustrated in previous research [49], in addition to their role in maintaining cell membrane integrity and stability, which results in blocking of free radicals release; therefore, improving the structure and function of the liver [52]. On the other hand, the current study showed a significant improvement in lipid peroxidation through a decline in the level of TBARS along with a marked restoration in hepatic GPx activity after cotreatment with *C. myrrha*. Our findings are in harmony with Seifried et al. who reported that there was a high association between the intake of antioxidant products and inhibited the occurrence of oxidative injury and inflammation [47]. Additionally, the anti-inflammatory properties of *C. myrrha* extract have also been demonstrated by Su et al., which illustrate the effective impact of ingredients present in *C. myrrha*. In research performed on mice, the *C. myrrha* extract led to the attenuation of paw edema caused by formalin along with its anti-inflammatory and analgesic activities [53]. At the same time, there were no significant differences in hepatic SOD, CAT, GSH, and TAC values after cotreatment with *C. myrrha* compared to ethanol intake only, where their values were slightly increased. The lack of significant elevations in the activity of antioxidant systems parameters that remove H_2_O_2_ could suggest an adaptive mechanism intended to keep sufficient H_2_O_2_ levels for cellular signaling [10].

In our research, a significant quantity of sesquiterpenoids in *C. myrrha* extract has been revealed. Among the sesquiterpenoids was curzerene which represented the highest percentage (31.07%) in *C. myrrha* extract. Curzerene is a sesquiterpenoid with antioxidant and free radical scavenging activities. It is effective against diseases caused by oxidative injury [54], in addition to other biological activities of curzerene and other sesquiterpenoids such as antitumor and antimicrobial properties [55]. Racine and Auffray reported that *C. myrrha* showed strong scavenging action to O₂^−^ radical. The research attributed this ability to the reaction between O₂^−^ radical and the furan ring of sesquiterpenoids in *C. myrrha* extract [56]. On the other hand, Xu and others demonstrated that the furan ring of sesquiterpenoids in *C. myrrha* has also exhibited DPPH radical scavenging activity, thereby suppressing cell death in the neural cell line [57]. Another major component identified in *C. myrrha* extract was hepta-2,6-dienoic acid (18.97%), which is a fatty acid and acts as an anti-inflammatory agent used for the treatment of the illness linked with TNF-α inhibition [58]. All of these reported activities of *C. myrrha* can be taken into consideration to confirm its therapeutic efficiency against ethanol toxicity in our study and may explain the significant improvement in histopathological and biochemical alteration which we recorded.

According to the previous studies, we found that there are several reasons why *C. myrrha* may be an efficient antioxidant, one being its content of polyphenolic compounds, which play a critical role in attenuating ROS production by inhibiting the activities of CYP 2E1, ADH, and xanthine oxidase (XO) that participated in the ethanol metabolism in the hepatic tissue [59]. Additionally, curzerene and other sesquiterpenoids are considered antioxidant agents due to their ability to eliminate free radicals, suppress ROS production, and inhibit mitochondrial programmed death through the reaction of a furan ring with radicals, thereby contributing to protecting tissue from ethanol toxicity [58]. A second potential pathway of lipid peroxidation suppression is the blocking of oxidative promotion via inhibiting the pathways of enzyme-dependent lipid peroxidation, such as the cyclooxygenase and lipoxygenase pathways [60]. A third potential pathway may be the capacity of *C. myrrha* and its components to enhance the production of natural antioxidants that possess ROS-scavenging activities [49]. All these potential antioxidant mechanisms of *C. myrrha* may explain the efficiency of this extract in attenuation of the toxic effects of ethanol such as inflammation, fat changes, and necrosis; therefore, improving the structure and function of the liver. Therefore, *C. myrrha* extract is a promising protective and therapeutic supplementation against toxic agents that may cause severe deterioration of body health.

## 5. Conclusions

In summary, ethanol consumption at a dose of 3 mL/kg for 30 days induced: (i) a significant elevation in liver function parameters and hepatic tumor markers, which are major biomarkers of liver injury and toxicity; (ii) a reduction in the antioxidant activities along with promoting lipid peroxidation by releasing ROS and RNS; (iii) histopathological alterations in hepatic tissues of rats, which reflect mechanisms of cellular injury. While supplementation with 500 mg of *C. myrrha* alone was safe on the liver structure and function. Moreover, cotreatment with *C. myrrha* in combination with ethanol seemed to have protective impacts on the liver, which were represented by attenuation of lipid peroxidation, reduction of the biomarkers level of both liver function and hepatic tumor, and improvement of the histological pattern of the liver due to its rich sesquiterpenoids content which possesses antioxidant properties and ROS-scavenging activities. At the same time, this cotreatment was not able to significantly restore the values of hepatic SOD, CAT, GSH, and TAC, which may suggest an adaptive mechanism for cellular signaling reasons. In this study, our findings will open novel perceptions for the utilization of *C. myrrha* alone or in combination with chemotherapeutic drugs to prevent or ameliorate the hazards associated with exposure to the different toxic agents.

## Figures and Tables

**Figure 3:**
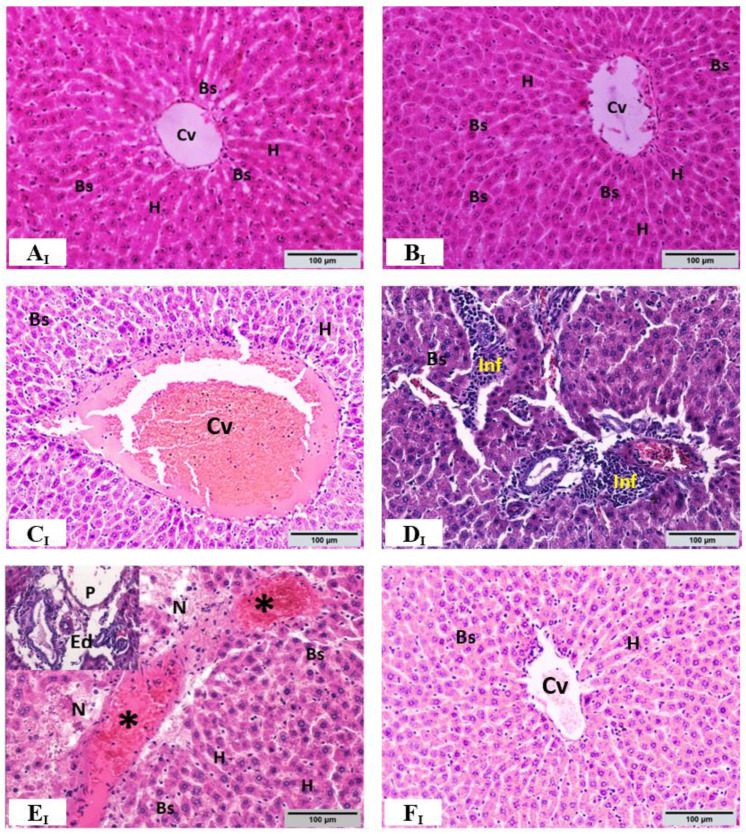
**I.** Representative photomicrographs for histopathological changes in hepatic tissues after 30 days of treatment. (**A_I_**): Liver of control rats showing normal hepatic architecture with typical histological structure of the hepatic lobule with normal central vein (Cv). Normal hepatic strands (H) radiate from the central vein towards the periphery of the hepatic lobule and are separated by sinusoidal spaces (Bs); and absent of any abnormal changes. (**B_I_**): The liver of rats from *C. myrrha* extract group showing no significant alterations in lobular structure. (**C_I_**–**E_I_**): Livers of rats from ethanol-treated rats show dilation of congested central vein, lymphocyte infiltration (Inf), sinusoid hemorrhage (*), necrosis (N), and “edematous (Ed) area around the blood vessels at the portal space (P) inset at the upper left corner of the figures (**E_I_**)”. (**F_I_**): Group treated with *C. myrrha* extract + ethanol showing improved histopathological alterations in hepatic tissues compared with ethanol-treated group. Hematoxylin and eosin (H & E stain; 200×, scale bar 100 µm).

**Figure 3:**
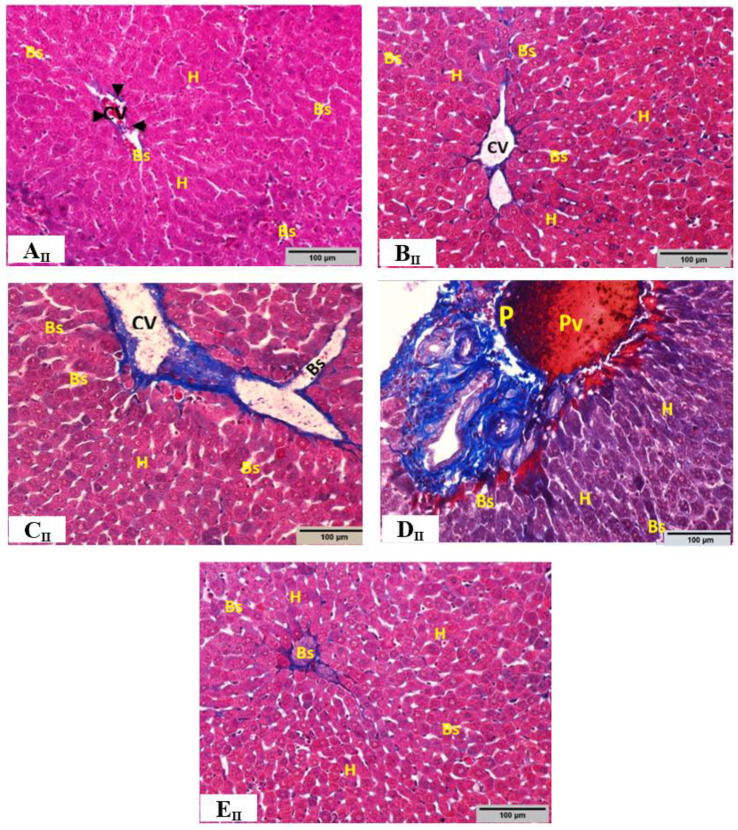
**II.** Representative images of Masson’s trichrome staining sections for liver tissues of control and experimental groups. (**A_II_**,**B_II_**): The hepatic tissues of control and *C. myrrha* extract-treated rats; respectively; showed a normal distribution of collagen fibers (blue) in sinusoidal spaces (Bs), around the central vein (Cv) which was bounded by an intact endothelium (arrowheads), and between parallel cords of hepatocytes (H). (**C_II_**,**D_II_**): The hepatic tissue of ethanol-treated rats showed a high deposition of collagen fibers (blue) around the central vein (Cv), dilation and congestion of portal vein (Pv) with portal spaces (P) expanded by abundant fibrous tissues (blue) as a marker for severe periportal fibrosis. (**E_II_**): Group treated with *C. myrrha* extract + ethanol showed noticeably reduced periportal fibrosis (blue). (200×, scale bar 100 µm).

**Table 1:** GC–MS analysis of phytochemical contents in *C. myrrha* extract.

Retention Time (Minute)	Phytochemical Contents of Myrrh Resin Extract	Area %	Area
13.17	3-methyl-6-(1-methylethylidene)-cyclohexene	0.44	848,956
14.03	Beta-elemene	0.12	226,618
14.63	Trans-caryophyllene	0.12	226,618
14.74	Gamma-elemene	0.84	1,635,662
15.32	CIS-.alpha.-bisabolene	0.16	302,442
15.79	Germacrene-D	0.56	1,092,838
16.02	Curzerene	31.07	60,172,180
16.91	4-ethenyl cyclohexanemethanol	0.14	262,910
17.16	Germacrene b	1.96	3,800,492
17.38	Menthofuran	2.21	4,272,107
18.19	3A,4,4A,7,7A,8,9,9A-octahydro-1,4,8-metheno-1H-cyclopent[f]azulene	13.20	25,573,312
18.31	Myrtenal	3.07	5,943,514
18.70	Aromadendrene 2	2.07	4,004,514
18.94	5-isopropylidenetricyclo [5.2.1.0(2,6)]dec-3-ene	2.75	5,317,004
19.15	7-cyclopentylidene-2-oxabi cyclo [4.1.0]heptane	0.51	991,674
19.37	Hepta-2,6-dienoic acid	18.97	36,733,940
20.14	(-)-Lepidozenol	0.13	249,712
21.37	Alpha thejenal	12.13	23,488,962
22.40	8-isopropenyl-9-isopropyltetra cyclo[4.4.0.0(1,5).0(2,6)]deca-7,9-diene	4.65	9,005,438
23.67	3,3-epoxymethano-6-(3’-isopropenyl cyclopropen-1’-yl)-6-methyl-2-heptanone	0.36	696,775
23.80	(-)-(e)-trans-bergamota-2,12-dien-14-al	0.20	386,455
24.11	(3z,6z)-dodeca-3,6-dien-1-ol	0.30	581,867
25.58	3,3-epoxymethano-6-(3’-isopropenyl cyclopropen-1’-yl)-6-methyl-2-heptanone	1.39	2,701,007
26.61	Lyratyl acetate	0.20	395,513
27.95	3-O-acetyl-6-methoxy-cycloartenol	0.16	307,148

**Table 2:** Effects of *C. myrrha* extract (500 mg/kg) and/or ethanol (40%, 3 g/kg) on lipid peroxidation biomarker and antioxidant activity in hepatic tissue after 30 days of treatment. TBARS, thiobarbituric acid-reactive substances; SOD, superoxide dismutase; CAT, catalase; GSH, reduced glutathione; GPx, glutathione peroxidase; TAC, total antioxidant capacity.

	Control	*C. myrrha*	Ethanol	Ethanol + *C. myrrha*
TBARS (nmol/g)	66.50 ± 2.09	66.28 ± 2.15	114.94 ± 3.82 ****	83.65 ± 2.87 ^####^
SOD (U/g)	68.68 ± 2.26	67.30 ± 2.67	44.24 ± 2.41 ****	48.30 ± 3.35
CAT (U/g)	6.22 ± 0.19	6.15 ± 0.13	4.11 ± 0.22 ****	4.67 ± 0.20
GSH (mg/g)	13.34 ± 0.52	13.57 ± 0.50	9.68 ± 0.45 ****	10.56 ± 0.53
GPx (U/g)	20.52 ± 0.84	21.59 ± 0.61	13.39 ± 0.65 ****	16.56 ± 0.52 ^#^
TAC (µM/g)	48.64 ± 1.66	52.73 ± 1.82	35.52 ± 2.26 ****	41.61 ± 1.45

All values are expressed as mean ± standard error (SE) of *n* = 7. **** *p* < 0.0001 vs. control; ^#^ *p* < 0.05; ^####^ *p* < 0.0001 vs. ethanol-treated group.

## Data Availability

Data are contained within the article.

## Supplementary Materials

The following supporting information can be downloaded at: https://www.mdpi.com/article/10.3390/toxics10120729/s1, Figure S1: Representative photomicrographs for histopathological changes in hepatic tissues of control and *C. myrrha* rats, Figure S2: Representative photomicrographs for histopathological changes in hepatic tissues after 30 days (A) and 45 days (B) of treatment with 40% ethanol.
