# Supplementary material for: Effect of Natural Commiphora myrrha Extract against Hepatotoxicity Induced by Alcohol Intake in Rat Model
Source: Toxics. 2022 Nov 26;10(12):729. doi: 10.3390/toxics10120729 (PMC9786033; doi:10.3390/toxics10120729)
Supplement: Supplementary file 1 [file toxics-10-00729-s001.zip › toxics-2022582-supplementary.pdf]

Supplementary Materials

(Preliminary Experiments)

1. Objective

Preliminary trials were performed to find out whether *C. myrrh* extract is safe on liver tissue and has no side effects. And also to verify the appropriate treatment conditions of ethanol to cause hepatotoxicity without mortality in rats.

1. Methods

25 adult male Sprague-Dawley rats (supplied by the animal house of the Science College at King Khalid University, Abha, Saudi Arabia) were used in this experiment. Before performing experiments, rats were acclimatized under standard laboratory conditions humidity (45 ± 5 %) and temperature (22 ± 2 °C) with 12 h light-dark cycles for 14 days with free access to water and normal food. The experiment was carried out according to the guidelines of the Institutional Animal Ethics Committee of King Khalid University, Saudi Arabia (approval No. 2022-2125). The experimental rats were randomly categorized into five groups with five animals in each group as follows:

a) The first group received normal water and diet as control;
b) The second group orally received 40% ethanol (3 g/kg) for 30 days;
c) The third group orally received 40% ethanol (3 g/kg) for 45 days;
d) The fourth group orally received *C. myrrha* extract (500 mg/kg) for 30 days;
e) The fifth group orally received *C. myrrha* extract (500 mg/kg) for 45 days.

During the treatment period, we recorded the mortality rate and any behavior changes. After 24 hours from the end of the dosage, the rats of all groups were sacrificed under light ether anesthesia. The liver samples were collected and washed in normal saline, then immediately fixed in 10% formalin for 72 h. Then, the samples were dehydrated in graded series of alcohol and cleared in two changes of xylene. After that, the samples were infiltrated in liquefied paraffin wax, then embedded in paraffin. Using a rotatory microtome, each sample was cut into 5 μm thick sections. To examine the liver structure and histopathological alteration in hepatic tissue, sections were stained with hematoxylin and eosin, then scanned under a digital light microscope.

1. Results

1.1. Effects of C. myrrha on rats

The rats of the control, *C. myrrha* (30 days), and *C. myrrha* (45 days) group showed normal behavior of movement and activities. However, the administration of *C. myrrha* for 45 days induced diarrhea and vomiting.

Regarding the histological examination, the hepatic tissue of rats after 30 days treated with *C. myrrha* extract showed an apparently intact architecture similar to that of the control group with no significant histopathological changes (Figure S1 A & B). While the treatment with *C. myrrha* extract for 45 days led to slight histopathological changes in the hepatic tissue, including lymphocyte infiltration and necrosis in some areas (Figure S1 C). So we found that the treatment with 500 mg/kg of *C. myrrha* for 30 days is appropriate for our study.

1.1. Effects of ethanol on rats

The administration of ethanol for both 30 and 45 days led to histopathological changes in the hepatic tissue, including dilation of the congested central vein, lymphocyte infiltration, necrosis, and the edematous area around the blood vessels at the portal space (Figure S2 A & B). On other hand, no deaths were recorded in the rats treated with 40% ethanol for 30 days, while two deaths

were recorded on days 40 and 44. Therefore, we suggested that the treatment with 3 g/kg of 40% ethanol for 30 days is appropriate for our study.

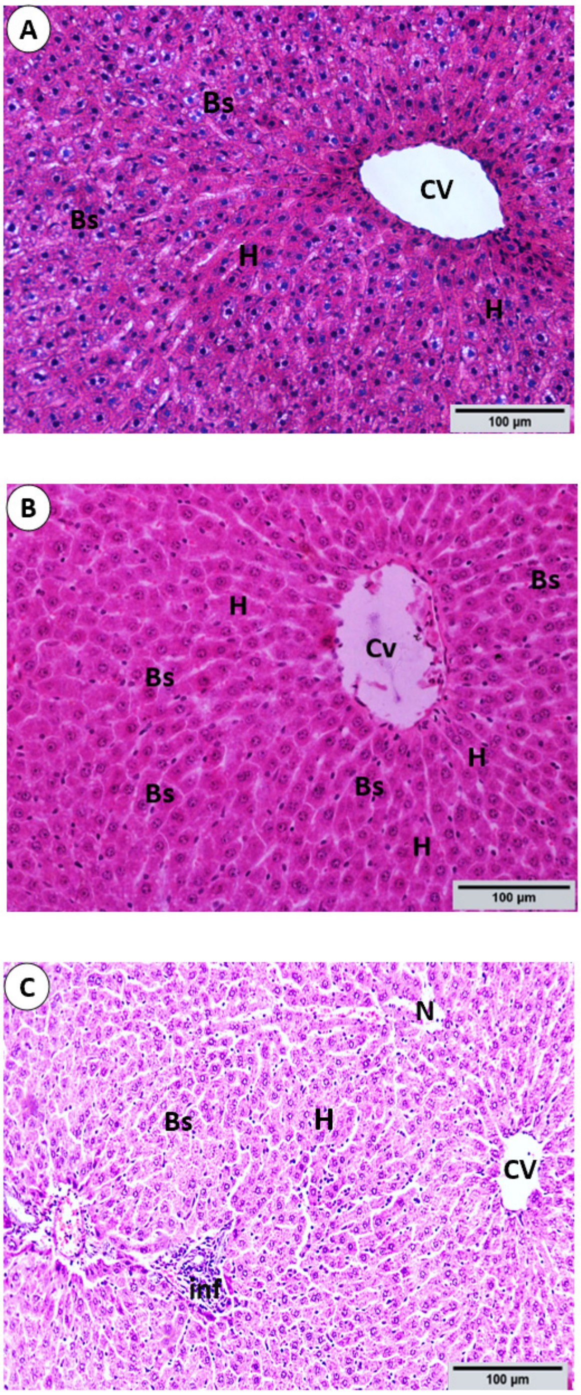

Figure S1: Representative photomicrographs for histopathological changes in hepatic tissues of control and *C. myrrha* rats. **(A):** Liver of control rats showing normal hepatic architecture with typical histological structure of the hepatic lobule with normal central vein (Cv). Normal hepatic strands (H) radiate from the central vein towards the periphery of the hepatic lobule and are separated by sinusoidal spaces (Bs); and absent of any abnormal changes. **(B):** The liver of rats after being treated with 500 mg/kg of *C. myrrha* extract for 30 days showed no significant alterations in lobular structure. **(C):** The liver of rats after being treated with 500 mg/kg of *C. myrrha* extract for 45 days showed lymphocyte infiltration (Inf) and necrosis (N). (H&E stain; 200×, scale bar 100 µm).

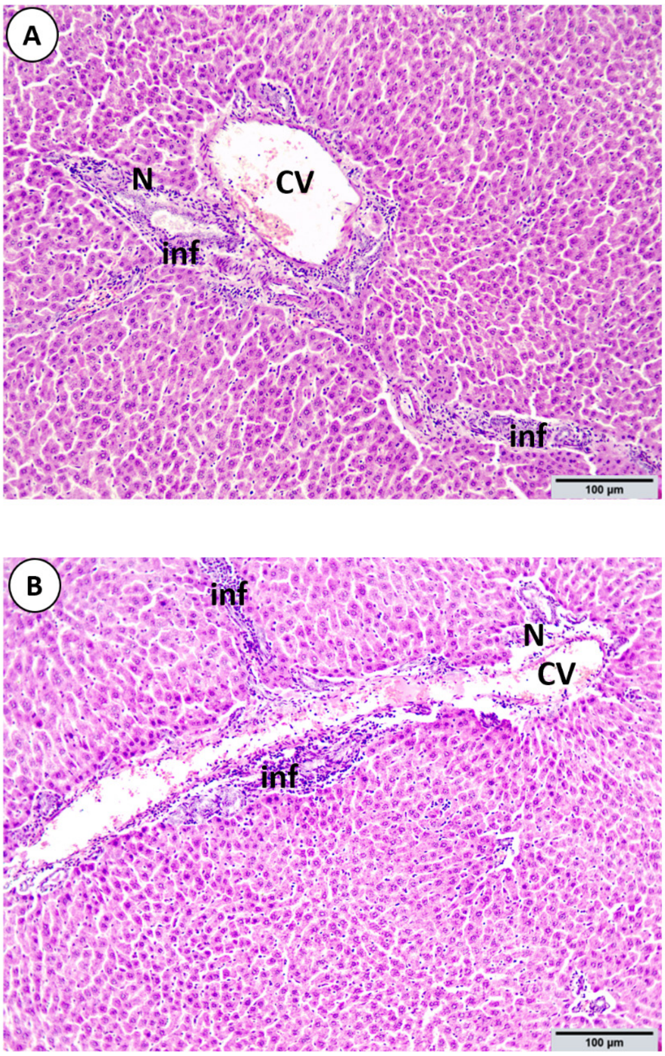

Figure S2: Representative photomicrographs for histopathological changes in hepatic tissues after 30 days **(A)** and 45 days **(B)** of treatment with 40% ethanol. Livers show dilation of congested central vein (CV), lymphocyte infiltration (Inf), and necrosis (N). Hematoxylin and eosin (H&E stain; 100×, scale bar 100 μm).
